# Immunotherapy in Advanced Non-small Cell Lung Cancer Patients: Ushering Chemotherapy Through the Checkpoint Inhibitors?

**DOI:** 10.7759/cureus.3254

**Published:** 2018-09-04

**Authors:** Bushra Kanwal, Sharmi Biswas, Robert S Seminara, Charan Jeet

**Affiliations:** 1 Lombardi Comprehensive Cancer Center, Georgetown University Medical Center, Washington DC, USA; 2 Pediatric, California Institute of Behavioral Neurosciences & Psychology, Fairfield, California , USA; 3 Neuroscience, California Institute of Behavioral Neurosciences & Psychology, Fairfield, USA; 4 Research, California Institute of Behavioral Neurosciences & Psychology, Fairfield, USA

**Keywords:** immunotherapy, immune checkpoint inhibitors, non-small cell lung cancer, chemotherapy, nsclc, pd-1 inhibitor, pd-l1 inhibitor, nivolumab, pembrolizumab, atezolizumab

## Abstract

New ways of exploiting the immune system for cancer treatment have been tested for decades with moderate outcomes. Based on previous immunotherapy knowledge, agents targeting immune checkpoints seem to be remarkably effective in a wide range of tumors. Immune checkpoint inhibitors in metastatic non-small cell lung cancer (NSCLC) provide longlasting responses in specific patients. Nevertheless, with overall response rates ≤ 20%, combinational protocols for various patient subgroups are needed. A good partner treatment to immunotherapy could be chemotherapy, as it successfully modulates the immune response either by controlling or enhancing the antitumor immune activity. Primary research provides promising results in metastatic NSCLC patients using this approach, but further large-scale trials are needed. The implementation of immunotherapy in nonmetastatic cases is also appealing. We review the potential clinical benefits of immunotherapy alone or in concert with chemotherapy in NSCLC.

## Introduction and background

Lung cancer accounts for significantly high rates of morbidity and mortality in the global population. Pulmonary or bronchial carcinoma, also known as lung cancer, is the most prevalent malignancy of the lower respiratory tract and it is divided into three major categories: non-small cell lung cancer (NSCLC), small-cell lung cancer (SCLC), and lung carcinoid tumors. NSCLC can be classified into three subgroups, including adenocarcinoma (which is the most common type), squamous cell carcinoma, and large-cell carcinoma [1]. Standard treatments, such as chemotherapy and/or radiation, although aggressive, are often inadequate for the elimination of tumor cells. This can be partially attributed to the high doses that are required damaging simultaneously normal tissues irreparably due to toxicity [2].

Current therapeutic protocols for NSCLC are based on chemotherapy regimens focusing on histology and targeted agents for patients with certain gene mutations [3]. The development of targeted cancer therapies has led to improved results in the metastatic setting for patients with lung adenocarcinomas which bear oncogenic driver alterations, such as epidermal growth factor receptor (EGFR) and re-arranged anaplastic lymphoma kinase (ALK) [4]. Nevertheless, even with those treatments, many patients with NSCLC do not achieve durable and stable disease control, and the survival rates are still low [5]. Therefore, it is of utmost importance to develop treatment designs that ensure prolonged disease control without serious adverse events.

Early attempts at nonspecific immunotherapy have shown mixed results. Tumor immune escape in lung cancer remains a hurdle to effective treatment. Low immunogenicity, antigen modulation, and tumor-induced immune suppression results in therapy resistance [4]. Despite the moderate initial outcomes of immunotherapy in NSCLC patients, innovative targeted immune-based approaches, including biological inhibitors, monoclonal antibodies, cells, vaccines, and genetic therapies, are underway as promising treatment alternatives. Such goals seem to be feasible, particularly after the successful implementation of targeted immune interventions, leading to the approval of this kind of regimen by FDA [4, 6]. The aim of the present review is to assess the therapeutic potentials of immunotherapy as a stand-alone treatment or in concert with conventional chemotherapy in NSCLC patients.

## Review

Lung cancer and therapeutical challenges 

Lung cancer is the second most common cancer after prostate cancer in men and breast cancer in women, demonstrating high rates of incidence and mortality. More than 85% of all lung cancers diagnosis are NSCLC [1]. The average survival of patients with untreated metastatic NSCLC is four to five months, with only 10% obtaining the one-year survival [7]. The standard-of-care first-line treatment for advanced NSCLC is platinum-based doublet chemotherapy. However, it is restricted to patients with a specific genomic profile who harbor no targetable mutations, such as altered EGFR and translocated or re-arranged ALK or ROS1. Nevertheless, even in this case, chemotherapy results in a poor response rate of 15 - 30%. Patients with progressed disease treated with taxane-based salvage chemotherapy also present a maximum 25% response rate [8].

A deeper understanding of the cancer-immunity circle has radically altered the therapeutic landscape of advanced NSCLC. Recently, immunotherapy with a new class of agents, checkpoint inhibitors, have demonstrated clinical responses with some patients experiencing longlasting, regression-free post-treatment periods. These monoclonal antibodies target either programmed cell death-1 protein (PD-1) or programmed death-ligand 1 (PD-L1), impeding immune escape. After encouraging evidence in 2015, nivolumab and pembrolizumab, two PD-1 checkpoint inhibitors, and in 2016, atezolizumab, a PD-L1 checkpoint inhibitor, received approval as second-line therapies of NSCLC. In 2016, pembrolizumab also was approved as a first-line treatment in NSCLC patients with high expression of PD-L1 [8].

Harnessing the immune system

Indeed, immunotherapy can have a leading role in cancer management either as a monotherapy or in combination with the standard treatments. To date, tumor immunotherapy spans a wide spectrum of modalities, from general activation of the immune response to targeted molecules against specific tumor antigens [9]. The greatest challenge is to ensure the maximum durable response with the minimum toxicity. Under this light, attempts are being made towards the generation of biological molecules that are able to regulate the immune response.

Nevertheless, the immune system becomes inadequate as the tumor progresses. The cancer immunoediting theory includes three stages: elimination, equilibrium, and escape. In the elimination stage, also called as immunosurveillance, malignant cells are successfully destroyed by the host’s immunity. However, tumor cells, which are less immunogenic, are able to escape immunosurveillance and reach the equilibrium stage. In the equilibrium stage, the immune system fails to completely eradicate all cancer cells, but it can effectively manage further tumor growth. In the escape stage, the tumor outgrowth is out of immune control, as the cancer cells that have escaped continue to proliferate [10].

It is evident that cancer cells co-opt specific pathways of the immune system, especially against T cells targeting specific tumor antigens, leading to tumor resistance. However, as a lot of checkpoints are generated by ligand-receptor interactions, they can be easily blocked by antibodies or regulated by engineered ligands or receptors [11]. Two of the most encouraging approaches involve the blockade of immune checkpoints through checkpoint inhibitors, such as cytotoxic T-lymphocyte-associated antigen-4 (CTLA-4) antibodies or PD1 inhibitors and vaccine therapy, which evokes specific immunity against certain tumor-related antigens [12].

Immune resistance and checkpoint inhibition

The antitumor immune response is a coordinated process initiated by the recognition of the tumor antigens by T lymphocytes followed by costimulatory binding of T-cell receptors (TCR) to peptide-major histocompatibility complex (MHC) on antigen presenting cells (APCs). CD28, a stimulatory molecule expressed on T cells, promotes T cell activation by binding to CD80 and CD86 (B7-1 and B7-2) ligands on APCs [13].

**Figure 1:**
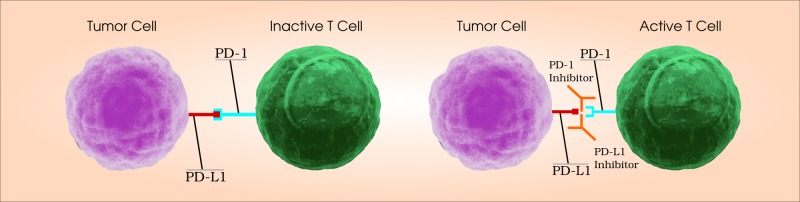
Role of programmed cell death protein 1 (PD-1) and programmed death-ligand 1 (PD-L1) pathway PD-1 is an immune checkpoint receptor on T cells and PD-L1 is a membrane protein expressed on tumor cells. Binding between PD-1 and PD-L1 inactivates the T cells. PD-1 inhibitors or PD-L1 inhibitors reactivate the T cells against tumor cells by preventing the binding between PD-1 and PD-L1.

On the other hand, PD-1 is an immune checkpoint receptor expressed on activated T cells. When PD-1 interacts with its ligands, PD-L1 and PD-L2, expressed on APCs on some normal and cancer cells, it results in T cell inactivation. Moreover, PD-L1 can interact with the B7 molecules, resulting in T cells turning off. PD-1-induced inhibition can happen peripherally in the tumors, suggesting a possible mechanism for adjusting immune resistance. Of note, the expression of PD-1 and PD-L1 seems to be promoted in malignant tissues compared to healthy ones in NSCLC patients as shown in Figure 1* *[14-15]. Thus, anti-PD-1 antibodies can be generated in order to bind to the PD-1 receptor, blocking its interaction with PD-L1/L2 and preventing T cell inactivation [16].

Immunotherapy as monotherapy

Based on the promising results of clinical trials since 2015, the United States (US) Food and Drug Administration (FDA) and the European Medicines Agency (EMA) have given their approval to nivolumab and pembrolizumab as second-line therapy. Nevertheless, pembrolizumab gained the approval as a first-line therapy for tumors with PD-L1 expression ≥ 50%. Recently, atezolizumab has also been approved for the management of heavily-treated patients with advanced NSCLC. Due to a lack of enough clinical trials comparing these drugs and because of their similar biological and toxicity profile, there is less possibility of recommending one treatment over the other [17]. Moreover, in 2017, the FDA, given the unmet medical need and based on the surrogate endpoints, allowed accelerated approval to pembrolizumab combined with pemetrexed and carboplatin for the treatment of patients with previously untreated NSCLC [18].

Such response, in combination with improved safety, has reignited the enthusiasm for testing these regimens in the first-line setting.

Nivolumab

The Phase I, multicohort Checkmate 012 study (NCT01454102) tested the efficacy and safety of nivolumab, a PD-1 immune checkpoint inhibitor antibody, as a stand-alone first-line treatment in 52 patients with advanced NSCLC. The outcomes were encouraging. The overall response rate (ORR) was 23%, including four ongoing complete responses (CR). More specifically, ORR reached 28% in patients with PD-L1 expression and 14% in patients with no PD-L1 expression. The 24-week progression-free (PFS) rate was 41%. The one-year OS was 73% and 18-month OS rate was 57%. Seventy-one percent of the patients experienced adverse events (AE), with fatigue being the most common (29%). In addition, serious AEs were observed in 19% of participants. Therefore, nivolumab could be considered a safe and effective option as first-line treatment in advanced NSCLC [19].

Pembrolizumab

In Phase III KEYNOTE 024 trial (NCT02142738), 305 patients with advanced and strongly PD-L1 positive NSCLC were recruited and randomized to the pembrolizumab group versus the standard of care platinum-based chemotherapy as the first-line treatment. The final results showed that pembrolizumab offered remarkably longer PFS and higher ORR with less toxicity than the platinum-based chemotherapy in this group of patients. In particular, the median PFS was approximately 10 months in the pembrolizumab group compared to six months in the chemotherapy group. At six months, the estimated OS rate was 80.2% in the pembrolizumab group, whereas in the chemotherapy group, this rate was calculated at 72.4%. The pembrolizumab group also outperformed chemotherapy in terms of RR (44.8% vs. 27.8%) and the median response duration. Lastly, pembrolizumab demonstrated a safer profile, as the prevalence (73.4% vs. 90.0% of patients) and severity of AE (26.6% vs. 53.3%) were significantly lower than chemotherapy [20].

Atezolizumab

The BIRCH study (NCT02031458) was conducted to test the effectiveness of atezolizumab, a humanized anti-PDL1 monoclonal antibody, in advanced NSCLC across three cohorts of therapy. For that purpose, 659 patients with a PD-L1 expression on ≥ 5% of tumor or tumor-infiltrating cells were recruited. After at least 12-months of follow-up, the ORR was 18% to 22% for the three lines of therapy with response maintenance, independently of the patients’ genomic profiles. All cohorts demonstrated comparable safety. Therefore, atezolizumab monotherapy can be considered an alternative option with good tolerability and improved efficacy in patients with PD-L1-selected advanced NSCLC. Moreover, the authors suggest that PD-L1 expression could act as a biomarker determining the patients that have a greater chance to benefit from this agent [21].

Immunotherapy in concert with chemotherapy

A large body of literature describes the regulatory effect that chemotherapy has on the immune response against malignancies and the ability of chemotherapy to promote PD-L1 expression in cancer cells [22]. Under this light, there is an increased interest for clinical evaluation of combinational therapeutic protocols, like immunotherapy and chemotherapy. Such a combination can serve binary purposes. First, checkpoint inhibitors could balance the immunosuppressive reactions of some chemotherapeutic drugs. Second, specific immunotherapeutic treatments could enhance the immunostimulating action of the chemotherapy [23].

Nivolumab and Chemotherapy

CheckMate 012, a Phase I, multicohort trial (NCT01454102), was designed, among others, to investigate the safety and efficacy of nivolumab in combination with conventional platinum-based doublet chemotherapy, as the first-line treatment in 56 patients with advanced NSCLC. The outcomes were promising. ORR for nivolumab, plus various chemotherapy regimens, ranged from 33 - 43%, while the 24-week PFS and two-year OS rates reached a maximum of 71% and 62%, respectively. Of note, those responses were obtained regardless of tumor PD-L1 expression, questioning the ambiguous role of PD-L1 expression as a predictive biomarker. As long as safety is concerned, no dose-limiting AEs were observed during the first six weeks of treatment. However, 45% of patients experienced severe toxicities and 21% of patients had to discontinue therapy due to treatment-related AE, a rate higher compared to monotherapy [24].

Pembrolizumab and Chemotherapy

The Phase II KEYNOTE-021 study (NCT02039674) compared pembrolizumab administration in combination with carboplatin-pemetrexed chemotherapy to chemotherapy alone. A greater number of patients with adenocarcinoma (97% versus 87%) were included in the combination group. The results were in favor of the combinational treatment, as the patients of this group demonstrated improved RR (55% versus 29%, with 80% RR among strongly PD-L1-positive tumors) and PFS (13 months versus 8.9 months) compared to those of the chemotherapy group. Nevertheless, the incidence of severe AEs was higher in the combination protocol (39% versus 26%). Remarkably, in the KEYNOTE-021 trial, the combination treatment minimized significantly the time to response (1.5 months versus 2.7 months), suggesting this approach could be beneficial for symptomatic patients. Those outcomes are in line with previous results in Phase I trials, proving that the immunotherapy, in concert with chemotherapy, can lead to improved management of NSCLC [25]. 

Atezolizumab and Chemotherapy

The GP28328 multicenter, multi-arm study (NCT01633970) evaluated the safety and efficacy of atezolizumab in combination with various first-line chemotherapy regimens in 76 patients with locally advanced or metastatic NSCLC. Regarding the safety profile, the most common treatment associated with serious toxicities were neutropenia (36 - 42%) and anemia (16 - 31%). In terms of efficiency, confirmed ORRs varied from 36 - 64% with a total of five incidents of CR. Median PFS ranged from 5.7 to 8.4 months, while the median OS exceeded 19 months in the group treated with carboplatin-pemetrexed-atezolizumab. Therefore, chemotherapy can be a valuable partner treatment to atezolizumab with significant and tolerable therapeutic effect [26].

## Conclusions

Innovative approaches generating checkpoint inhibitors have led to encouraging preliminary results and longlasting responses in NSCLC. The dramatic efficiency and safety outcomes from recent studies testing novel immunotherapy agents in resistant or metastatic NSCLC have increased the scientific interest in expanding their evaluation to a greater number of lung cancer patients. As a consequence, multiple clinical trials assessing integrated immunochemotherapy protocols are ongoing or in development. Their success depends to a great extent on the rationale combination of these two modalities based on safe preclinical data. Last but not least, the optimal dosing, the choice of the proper immunotherapy agent, the ideal combinational protocol, and patient selection are still to be determined in order to achieve the maximum efficacy with the minimum toxicity, improving, as a result, patients’ overall survival and quality of life.
